# Multidimensional scale of meaningful work: construction and validation

**DOI:** 10.3389/fpsyg.2025.1578825

**Published:** 2025-03-19

**Authors:** Aleksandra Batuchina, Inga Iždonaitė-Medžiūnienė, Rron Lecaj

**Affiliations:** SMK College of Applied Sciences, Klaipėda, Lithuania

**Keywords:** meaningful work, multidimensional scale, scale development, job design, meaningful leadership, organizational commitment, social impact, work-life balance

## Abstract

Meaningful work allows individuals to align their jobs with their personal values and passions, resulting in greater fulfillment and commitment. When work is meaningful, employees develop resiliency during challenging times, viewing challenges as opportunities rather than obstacles. However, there is no unified definition of meaningful work as different fields attribute different dimensions to the concept. Therefore, the evaluation and measure of meaningful work dimensions is important and should evolve in response to modern trends. The purpose of this paper is to introduce and validate the Multidimensional Scale for Meaningful Work. Following a structured scientific search on the acknowledged components of meaningful work, three studies were conducted on Content Validity, Response Process Validity, and Internal Structure Validity. Utilizing a mixed-methods approach, qualitative and quantitative data aided in the development and validation of this scale. The combined results of the studies showcase a unified measure assessing the five dimensions of Meaningful Work: *Job Design/Environment, Meaningful Leadership, Organizational Commitment, Work and Life Balance, and Social Impact.* Lastly, 80 items for all dimensions have been indicated and persevered throughout the rigorous analysis procedures. The scale provides a transformation of a subjective phenomenological concept onto quantitative measurable dimensions. Institutions that use the scale can more deeply understand their own organizational climate and intervene depending on which dimension is lacking. The scale has been designed to measure both the individual experience of the dimensions and the organizational experience.

## 1 Introduction

In the era of widespread information communication technologies, increasing job automation, and fading work and non-work boundaries, introspective questions about seeking meaningful work come forth. Meaningful work is yet to have its own definition or a full consensus; however, the general tenets still apply (Sandoghar and Bailey, 2023). In a series of publications, a network of academics and industry representatives revealed that meaningful work is more easily conceptualized on the tenets of positive significance or purpose, constituent components of meaningful work, how individuals and their work fit, and fulfillment through work (Sandoghar and Bailey, 2023). While positive significance is related to purpose and growth, constituent components of meaningful work rely on finding meaning with one’s work and growing, as a positive subjective experience (Martela and Pessi, 2018; Sandoghar and Bailey, 2023). Further, the fitness between individuals and their work points out that meaningful work is built on characteristics that individuals pursue in their work as an extension of their own values (Lührmann et al., 2024; Sandoghar and Bailey, 2023). Lastly, the definition of meaningful work through the idea of fulfillment relies on individuals attaining fulfillment through their work (Martela et al., 2021). Despite their differences, all of these definitions developed over the years center around the main theme of the subjective experience of work, and how these subjective experiences saturate meaning-making in work. This is supported by subjectivism and phenomenologists suggesting that finding meaning in one’s work and meaningful work are personal (Van Der Deijl, 2022). Therein, meaning-making and meaningful work are manifested differently for different people (Michaelson, 2021; Michaelson et al., 2014).

Although conceptualizations and frameworks may slightly differ for meaningful work, its benefits include increased motivation, organizational commitment, engagement, satisfaction, efficacy and performance, positive affect, work relationships, life meaning and satisfaction (Hu and Hirsh, 2017). Moreover, it significantly facilitates the relationship between emotional energy spent and job performance, enhancing employees’ productivity by fostering a deeper connection to their tasks and responsibilities (Rabiul et al., 2023). To add to that, it is also positively correlated to self-efficacy, job involvement, and proactive behavior (Kim, 2023). Consequently, meaningful work decreases burnout, stress, and counterproductive behaviors (Tan and Yeap, 2022).

In the current economic and labor market state, employers face challenges finding the right employee with the right skills, therein, making meaningful work an increasingly important pragmatic issue (Michaelson, 2021; Michaelson et al., 2014). With the integration of socio-philosophical concepts into the labor market, there are also challenges of meaningful and ethical leadership and the stances which organizations take toward employees (Vveinhardt, 2022). Thus, meaningful work as a psycho-social and phenomenological concept has also emerged in various organizations showing its positive effects on employee work engagement, work-life balance, and personal growth (Fairlie, 2011; Arora and Garg, 2024). The concept of meaningful work is usually used to evaluate the quality of work (Van Der Deijl, 2022). It is also related to the degree of meaning that employees believe their work has, with their personal values, and with their relationships with colleagues and leaders (Rosso et al., 2010). Scoping further, recent research results focus on the idea that employees seek not only professional growth, but also meaningful work and genuine connections integrated to their well-being (Miller, 2024). This trend indicates that modern work environments are correlated with an increased value for financial, emotional, and social dimensions (Monteiro and Joseph, 2023). Therefore, to retain, train and motivate employees, organizations are obliged to create conditions promoting the development of a meaningful work culture. To facilitate this, many researchers offer a wide range of strategies for enhancing organizational culture to foster meaningful work and employees’ adequate understanding of it (Miller, 2024; Pathiranage et al., 2021). Miller (2024) suggests that human resource strategies should align employee roles with organizational purpose and personal values while emphasizing the importance of technology to facilitate collaboration, increase trust and develop purpose-driven culture. In terms of work engagement contributions, there is a need for organizations to construct an environment that facilitates meaningful work to influence work engagement and effective commitment to the end of organizational transformation (Faisaluddin et al., 2024). Their research showed that work engagement acted as a mediator in the relationship between meaningful work and effective commitment to change.

Through a digitalization perspective, researchers argue whether this process has a positive impact on meaningful work, while others see it as a threat (Marsh et al., 2022; Bolli and Pusterla, 2022). On one hand, digitalization may result in work fragmentation, repetition, algorithmic management and diminished relevance of work using digital technologies that can impact its meaningfulness; On the other hand, digital technologies make work more meaningful by reducing tedious, repetitive tasks and creating more time for employee leisure (Arora and Garg, 2024). Considering the contradictory issues of digital technologies and meaningful work, Arora and Garg (2024) generalize previous research stating that digital technologies created a lack of work opportunities, leading to a crisis of meaninglessness. Still, work digitalization has increased access to meaningful work for people with disabilities. Hence, considering these conflicting findings and many important aspects of meaningful work facilitates conditions for its complexity.

Seeing the literature trends, meaningful work appears to be multidimensional including many areas or dimensions that are closely interconnected such as perceived social impact, leadership, and work environment (Iždonaitė-Medžiūnienė and Batuchina, 2023). It is also linked to other fields such as organizational behavior, organizational psychology, humanities, communication studies, and ethical implications (Blustein et al., 2023). Meaningful work dimensions are connected to employees’ working contexts which highlight contextual dimensions of meaningful work such as organization-specific, social context–related, job design–related, and employment-related working conditions (Blustein et al., 2023). Other research refers to meaningful work as a contribution to personal life purpose and its value and usefulness to others through work engagement, commitment, and job satisfaction, inclusive and meaningful leadership, and ethical leadership (Allan et al., 2019; Charles-Leija et al., 2023; Frémeaux and Pavageau, 2022; Mosquera et al., 2024; Shafaei and Nejati, 2024). Blustein et al. (2023) distinguish meaningful work impact at the organizational and individual level by stating that organizations tend to observe the increased in-role or extra-role in organizational citizenship behavior, employee creativity and innovation. These authors argue that growing research reveals the dark side of meaningful work which results in negative effects on employees’ well-being, work–family conflicts, and other personal sacrifices due to higher levels of work devotion and meaningfulness. Such opposite aspects emphasize the multiple contexts that frame meaningful work, which also is embedded in macro level, social, organizational, and job-related factors (Blustein et al., 2023).

The above scientific review of literature presents a holistic reflection of the meaningful work concept which still does not provide any unified understanding, thereby emphasizing the importance of meaningful work scales to be comprehensive, inclusive of different dimensions, and valid. It is clear that the reason behind the lack of a common definition of MW is its multidimensionality and subjectivity component (Both-Nwabuwe et al., 2017; Sandoghar and Bailey, 2023). However, there is a need for the presence of meaningful work within working environments and in individuals for the decrease of burnout, and increased motivation and perceived social impact. While meaningful work is becoming exponentially important, it is equally crucial to have the means of assessing this phenomenological concept (Iždonaitė-Medžiūnienė and Batuchina, 2023; Steger et al., 2012). Previous scales report on meaningful work, positive meaning of work, and other variables without taking into account perceived social impact (Lips-Wiersma and Wright, 2012; Steger et al., 2012). Other issues stand in the cultural differences both in-institution, between-institutional, and overall different international and intercultural issues (Lips-Wiersma and Wright, 2012). As such, the authors of this paper have constructed and aim to present the Multidimensional Scale for Meaningful Work (herein-after, MSMW). We acknowledge the work conducted previously by other researchers and practitioners in efforts to define this concept. Our methodology aimed to structure and outline the most supported existing dimensions of meaningful work so as to validate this draft of the MSMW through the following research questions:

Q1: What is the construction of the MSMW covering various dimensions?

Q2: How valid is the MSMW and its subscales?

## 2 Development of the MSMW

### 2.1 Construction of the MSMW

**Figure 1:**
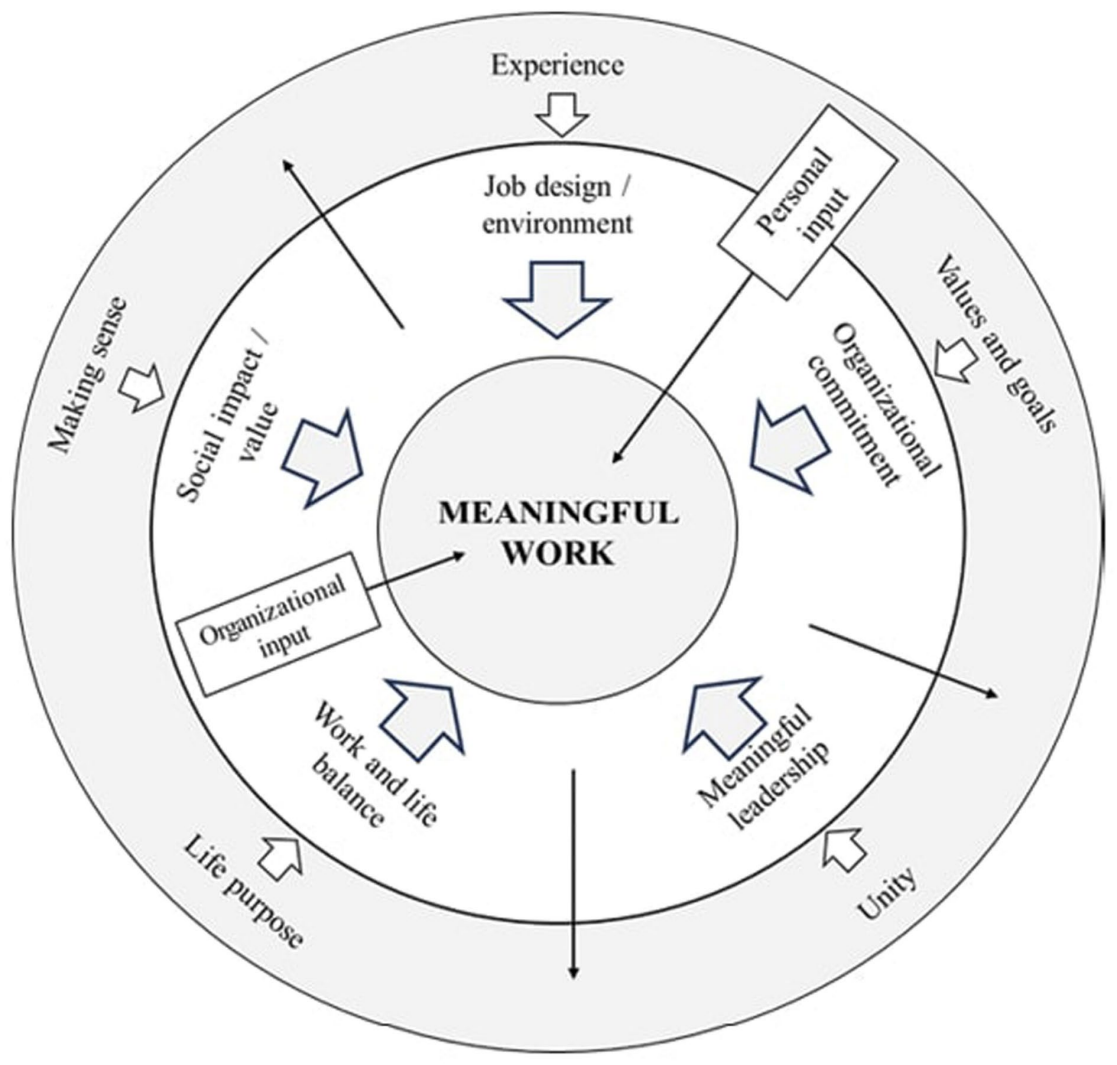
Multidimensional Framework of Meaningful Work” (Iždonaitė-Medžiūnienė and Batuchina, 2023, p. 69).

The MSMW was created based on the search of empirical studies examining meaningful work in organizational psychology, humanities, and other related disciplines. This search of empirical studies in English in scientific journals since 1960 was performed during the summer of 2023, using APA PsycInfo, PsycARTICLES, Academic Search Complete (EBSCO) Psychology Databases, and Google Scholars. The main search terms were “meaningful job/work” and other synonyms including “purposeful work/job,” “purpose-driven work,” “empowering work” and others. More than 11,000 publications were considered during the review of empirical and theoretical studies. Additionally, the review of MW scales aided in finding a distinction between the MSMW with existing ones. We used the final constructed meaningful work framework of 99 items based on previously conducted research analyses. The Multidimensional Meaningful Work framework was introduced by Iždonaitė-Medžiūnienė and Batuchina (2023), and focused on five major dimensions including *Job Design, Organizational Commitment, Meaningful Leadership, Work and Life Balance, and Social Impact* (see Figure 1).

## 3 Study 1—Content validity

The aim of this study was to provide qualitative evidence to the validity of the items of the MSMW. Utilizing qualitative in-depth interviewing, participants representing small and medium enterprises (SMEs) and academicians were involved in the validation of the content of the MSMW. Content validity ensures that the items are explored in-depth regarding the language use, translation, order and item construction (Lamm et al., 2020). Therein, its importance is highlighted as it provides a solid base for further piloting the MSMW.

### 3.1 Method

#### 3.1.1 Procedure and sample

Item comprehensibility was tested among 17 in-depth interviews – of which, there were Small and Medium Enterprises (*n* = 9) and academicians (*n* = 8). Several criteria were considered for the selection of the participants. For SME representatives, the criteria required them to be experts in the field of Human Resources, holding an HR position or directly being involved in the construction of the HR strategy of the enterprise. In addition, at least 5 years of experience were required in this field including participation in learning and training activities connected to employee and organizational wellbeing. For academicians, the criteria required the participation of PhD holders in fields such as education, behavioral psychology or similar, having at least one publication related to meaningful work, work satisfaction or other related topics. An additional criterion required research experience in the construction of psychometric questionnaires and tools.

Related to the analysis, research on data saturation analysis found that out of 15 concepts identified as most important in their qualitative research, 90% of them emerged with an *n* = 8 research sample, and 100% with an *n* = 14 sample; although, some researchers argue that data saturation is achieved through 12–30 interviews (Gugiu et al., 2020; Iždonaitė-Medžiūnienė and Preikšaitienė, 2024). Participants were provided with the MSMW prior to the interviews, and were asked to go through the items while highlighting confusion or inaccuracy. In-depth interviews lasted from 30 to 60 min, and included open-ended questions related to the MSMW. Business representatives and academicians were able to provide their expertise and further offer suggestions or provisions to be made. In the current context, in-depth interviews were audio recorded and note-taking occurred for the purpose of retaining information provided and the discussions occurring.

#### 3.1.2 Analysis

The qualitative research study was based on the data saturation principle, which ensures that the data collected is relevant and comprehensive. According to Naeem et al. (2024, p.1), saturation in qualitative research “denotes the stage at which the data collection and analysis have been exhaustively examined and comprehended, and no additional themes are emerging.” The items of the subscales were checked by expert interviews, consisting of business representative experts (*n* = 9) and academician experts (*n* = 8) to assess the content validity. Participants were provided with the definitions of each subscale to evaluate each item against three levels: completely representative, somewhat representative, and not representative (Bashir et al., 2022).

#### 3.1.3 Results

Business representatives and employees in academia provided their insights and recommendations per subscale. Initially, the scale contained 99 items and the study sought out to modify the existing items and dimensions. The initial scale contained the following:

1. *Job design/environment (19 items)*—the dimension was based on research by Morgeson and Humphrey (2006) and concentrates more on the inner sense of satisfaction of work, while leaving only the elements, referring to independence/autonomy, new opportunities, clarity, and variety of job duties, security, feedback, healthy competition, and decision-making.
2. *Meaningful leadership (23 items)*—the dimension was based on several researchers and practitioners and focuses on the modern employee that can contribute to their own meaningful work and the meaningful work of their employees (Arnold et al., 2000; Frémeaux and Pavageau, 2022; Houghton et al., 2012).
3. *Commitment to the organization (20 items)*—the dimension was based on the research conducted by Allen and Meyer (1990), Benkhoff (1997), Sheldon (1971), Salancik (1977), the concept of decent work (Blustein et al., 2023), and includes satisfaction, emotional attachment, care about the future of organization, loyalty while not omitting commitment-based aspects (Mortimer, 2023).
4. *Work and life balance (28 items)*—the dimension was based on research conducted by Agha et al. (2017), Hayman (2005), Warwick (2017) and concentrates on work interference with personal life, personal life interference with work, work-personal life enhancement, health and stress-coping strategies. Moreover, job contribution to one’s life purpose was also encapsulated in this dimension (Charles-Leija et al., 2023).
5. *Social impact (9 items)*—the dimension was based on the research conducted by Grant (2008), Grant and Campbell (2007), Fairlie (2011), Izquierdo and Pérez (2022), and focuses on the social impact on colleagues, clients/customers, people outside the organization, and global impact.

As can be seen on Table 1, minimal changes are recorded, but the data revealed commonalities between academicians and business representative responses. Similar responses between the different categories of experts in specific items resulted in item modification or deletion at the authors’ discretion. After the content validity implementation and revision of the questionnaire, 84 items were chosen for the stage of response process validity.

**Table 1:** Results of data saturation (Study 1).

Subscale title	Business representative recommendations (*n* = 9)	Academician recommendations (*n* = 8)
Job design/environment (19 items)	Experts recommended changing the formulation of the item “*The job itself provides feedback on my performance.”* Recommendations included changing the word *head/boss* (of the department) to *leader/ manager.*	The scale “*The job allows freedom, independence, or discretion in work scheduling, sequence, methods, procedures, quality control, or other decision making”*, was seen to be broad and repetitive.
Meaningful leadership (23 items)	The item *“I explain how my work group fits into the company”* was considered a repetition, and was deleted. The item “*I try to mentally evaluate the accuracy of my own beliefs about situations I am having problems with”* was considered unclear, however, the authors decided to subject it for further analysis before deletion.	One academic proposed connecting this set of items with the Multifactor Leadership Questionnaire (Avolio and Bass, 2004), but the authors will consider it later on.
Commitment to the organization (20 items)	The formulation of this item was unclear: *“I am dedicated to this organization because I fear what I have to lose in it”*, as a result, it was changed to “*My dedication to this organization is based on the fear of losing my job*.” The item *“I often feel anxious about what I have to lose with this organization”* was reformulated to *“I often feel anxious while working at the organization.”* Also, the item: “*I feel it is morally correct’ to dedicate myself to this organization”* was not clear. As a result, the authors removed it.	Recommendation was given to change reverse items, such as *I do not feel like ‘part of the family’ at my organization*. The item *“I often feel anxious about what I have to lose with this organization”* also was unclear and, as a result, it was deleted.
Work and life balance (28 items)	The item *“I am satisfied with the overall work situation”* was considered misleading. The authors changed it to *“I am satisfied with overall workload and character.”* The suggestion was to reformulate all the reversed items into positive meanings.	The suggestion was to reformulate all the reversed scales into positive meanings, such as: “*At work I am under such pressure that I have no time to do things properly”;* “*There is rarely a day at work when I am not stressed and overworked”; I do not have the strength for new activities and tasks; My job makes personal life difficult”; I am unhappy with the amount of time for non-work activities.*
Social impact (9 items)	The item “*Even if I do not like particular organizational changes*, *I comply with those policies if they contribute to the continuous prosperity of the institution”* was considered unclear, and was deleted. The item *I am very aware of the ways in which my work is benefiting customers/ was specified* with term customers and target groups. The suggestion was to add open questions after every group scale to have an opportunity to explain and give examples.	The scale: The work performed on the job has a significant impact outside the organization was detailed by: “The work performed on the job has a significant impact on people outside the organization. The suggestion was to add an opportunity to choose to the Likert scale: *I do not know*, to measure the informativeness of the workers.

## 4 Study 2—Response process validity

The second study’s aim was to further confirm the validity of the MSMW through quantitative measures. Through a small sample of 65 respondents, the scale was distributed and further tested for validity and comprehension undergoing response process validity.

### 4.1 Method

#### 4.1.1 Procedure and sample

In a construction company with an estimated number of 148 employees, 68 employees agreed to become involved with the MSMW. However, in analyzing the quantitative data, this number of employee responses was reduced to 65 (see Table 2). While the MSMW had been constructed in English, it was translated and adapted to Lithuanian for this sample. The questionnaire consisted of the 5 subscales with a 5-point ranked Likert scale. In total, 84 questions from these 5 scales were presented in this version. Additional demographic questions were included in the questionnaire, such as: department, education level, work experience, age. Additionally, questions such as “*Rate from 0 to 10 how likely you are to recommend the “Title of the Organization” as an employer to your friends or acquaintances?*” were added at the request of the company’s management. Additional open questions were added at the end of the questionnaire to collect recommendations and insights from the respondents for the questionnaire improvement. The survey was performed in accordance with social research ethics rules: anonymity, confidentiality, voluntary participation in research and others (Badampudi et al., 2022).

**Table 2:** Socio-demographic information (Study 2).

Category		Frequency	Percentage
Age	From 21	0	0
	22–25	4	6.2
	26–30	7	10.8
	31–40	39	59.9
	41 and older	15	23.1
Number of years in the COMPANY	Less than a 1 year	15	23.1
	1–2 years	9	13.8
	2–3 years	3	4.6
	More than 3 years	38	58.3
Whether you have subordinate workers	Yes	34	52,4
	No	31	47.6
Education	High school	7	10.8
	VET	11	16.9
	Higher education	46	70.8
	Other	1	1.5

### 4.2 Analysis

To complete the response process validity all items that were unclear were revised and tested. Several perspectives were considered as important to response process validity according to Lamm et al. (2020):

1. From the functional perspective, the authors of this instrument ensured that consent and information procedures took place prior to research conduction.
2. From the international perspective, the authors of this instrument considered translation and localization issues as they translated the scale from English to Lithuanian to adhere to the Lithuanian context of these studies.
3. From the research participants’ perspective, they had a possibility to indicate whether the requested response was clear or if there was the case of any misunderstanding while providing their responses.

### 4.3 Results

Through quantitative descriptive analyses, the items were checked for their comprehensibility and response process validity coupled with demographic data. Simultaneously, a simple coding and grouping analysis occurred qualitatively to categorize open-ended responses. The research participants indicated whether they understood the requested response, highlighted all confusions with the response provision in connection with all subscales, and all responses to unclear instrument items were analyzed consequently.

## 5 Study 3—Internal structure validity

After the first two studies, it was important to disseminate the MSMW in a larger sample of employees while it contained all the revised items. Therein, using a sample of 390 employees from the construction field, the MSMW was tested for its *Internal Structure Validity*.

### 5.1 Method

#### 5.1.1 Procedure and sample

To test the factor structure and validate the scale, an exploratory factor analysis was performed. Thus, after performing revisions, a questionnaire with 84 questions on 5 scales was provided to another organization (working in construction). In total 390 responses were received (450 questionnaires were distributed, 390 responses were received), however a ratio of five respondents per item is considered to be an eligible number to perform factor analyses (Lamm et al., 2020). Within the given context, 390 is considered to be a reasonable number for the analysis since accurate estimates of population parameters can be obtained with samples as small as 100 (Fabrigar et al., 1999).

#### 5.1.2 Analysis

At this stage several analyses within SPSS were conducted including: descriptive statistics, reliability analysis, and an exploratory factor analysis (EFA) was completed, and *Cronbach alpha* coefficient was indicated for each of the subscale, and the scale in general. EFA and scale development guidelines were closely followed as instructed in scale development articles (Carpenter, 2018).

#### 5.1.3 Results

Demographic information showed diversity within the sample for this study relating to age, education level, number of years within the company. Most of the participants of this study were aged *21–25* (24%) and *41 and older* (22%), and a majority of participants were *Vocational Education and Training (VET)* graduates (47%). There was also variety among participants that had Participants reported varying lengths of employment with the company, with 31% indicating they had been with the company for 1–2 years, and 29% for 3 years or more (see Table 3).

**Table 3:** Socio-demographic Information (Study 3).

Category		Frequency	Percentage
Age	From 21	81	21
	22–25	94	24
	26–30	74	19
	31–40	57	15
	41 and older	84	22
Number of years in the company	Less than a 1 year	75	19
	1–2	121	31
	2–3	81	20
	More than 3 years	113	29
Whether you have subordinate workers	Yes	127	33
	No	263	67
Education	High school	86	22
	VET	183	47
	Higher education	91	23
	Other	30	8

Moreover, a reliability analysis was conducted to assess the internal consistency of the scale and subscales. The results demonstrated that all scales were reliable measures. To scope further, Job Environment/Design assesses factors such as job duties, clarity and diversity of tasks, autonomy in performing tasks, sense of security, and decision-making capabilities. The Cronbach’s alpha coefficient was 0.887, with a scale average of 4.07. Meaningful Leadership assesses aspects such as the provision of personal and professional support, attitudes toward community members, work and tasks, expression of community spirit, and the development of work-friendly relationships. The Cronbach’s alpha coefficient was 0.897, with a scale average of 4.16. Further, Organizational Commitment measures personal commitment to the organization’s community, identification with the organization, pride in the organization, and the desire to remain in the community despite alternative options. The Cronbach’s alpha coefficient was 0.838, with a scale average of 3.56. Work-Life Balance evaluates the interference of work duties with personal life and vice versa, the complementarity of work and personal life, and the ability to balance work and personal life to improve quality of life. The Cronbach’s alpha coefficient was 0.828, with a scale average of 3.06. Lastly, Social Impact/Contribution investigates the impact on the organization’s community and interested groups (e.g., customers, partners), the manifestation of impact outside the organization, and striving for the greater good. The Cronbach’s alpha coefficient was 0.846, with a scale average of 4.01. The item-total correlations (*r/itt*) for all items were greater than 0.70. Therefore, the deletion of any item would result in a lower Cronbach’s alpha coefficient for the remaining items, confirming the robustness of the scales.

Later, data were tested for univariate normality. The Kaiser-Meyer-Olkin (KMO) measure of sampling adequacy was 0.897, indicating that the sample was appropriate for factor analysis. Furthermore, the findings revealed a five-factor solution, which explained 60% of the variance. These indicators suggested that the correlation matrix was suitable for factor analysis. Additionally, an analysis of the rotated component matrix coefficients for each variable showed that not all items exceeded the threshold of 0.60. Consequently, it was deemed appropriate to exclude some items from the scale. Specifically, the following items were removed: *“The job allows me to make decisions about what methods I use to complete my work”* (Job Design); *“I worry about the loss of investments I have made in this organization”* and “*I feel it is morally correct’ to dedicate myself to this organization”* (Commitment to the Organization); and *“I try to mentally evaluate the accuracy of my own beliefs about situations I am having problems with”* (Meaningful Leadership; see Table 4).

**Table 4:** Rotated component matrix.

Items	Item acronym	Components				
		1	2	3	4	5
Job design/environment	JDE1	0.786				
	JDE2	0.758				
	JDE3	0.756				
	JDE4	0.747				
	JDE5	0.745				
	JDE6	0.745				
	JDE7	0.742				
	JDE8	0.737				
	JDE9	0.734				
	JDE10	0.734				
	JDE11	0.723				
	JDE12	0.722				
	JDE13	0.715				
	JDE14	0.712				
	JDE15	0.709				
	JDE16	0.7				
	JDE17	0.699				
	JDE18	0.695				
	JDE19	0.595				
Commitment to the organization	CO1		0.887			
	CO2		0.84			
	CO3		0.835			
	CO4		0.797			
	CO5		0.745			
	CO6		0.745			
	CO7		0.743			
	CO8		0.737			
	CO9		0.734			
	CO10		0.732			
	CO11		0.731			
	CO12		0.722			
	CO13		0.72			
	CO14		0.719			
	CO15		0.712			
	CO16		0.707			
	CO17		0.648			
	CO18		0.591			
	CO19		0.543			
Meaningful leadership	ML1			0.816		
	ML2			0.808		
	ML3			0.802		
	ML4			0.799		
	ML5			0.798		
	ML6			0.78		
	ML7			0.778		
	ML8			0.767		
	ML9			0.754		
	ML10			0.742		
	ML11			0.742		
	ML12			0.731		
	ML13			0.725		
	ML14			0.722		
	ML15			0.719		
	ML16			0.717		
	ML17			0.698		
	ML18			0.698		
	ML19			0.688		
	ML20			0.688		
	ML21			0.62		
	ML22			0.512		
Work and life balance	WLB1				0.778	
	WLB2				0.762	
	WLB3				0.739	
	WLB4				0.778	
	WLB5				0.762	
	WLB6				0.739	
	WLB7				0.739	
	WLB8				0.659	
	WLB9				0.653	
	WLB10				0.625	
	WLB11				0.613	
	WLB12				0.61	
	WLB13				0.601	
	WLB14				0.601	
Social impact	SI1					0.878
	SI2					0.867
	SI3					0.762
	SI4					0.741
	SI5					0.72
	SI6					0.715
	SI7					0.703
	SI8					0.701
	SI9					0.657
	SI10					0.654

## 6 Combined results

We present the matrix of the scale in Table 5 highlighting the scale dimensions, number of items for each and item description. We grouped all items under each dimension into smaller item groups. As the work environment is going to be evaluated, we suggest items about opportunities, job performance, and job security and feeling good. Evaluation of organizational commitment will go through employee feelings about the organization and inside the organization as well as organizational loyalty and personal connection with the organization. We suggest meaningful leadership as a dimension to be evaluated through items about employee relations with own supervisors, employee personal goal management, teamwork, and relations with own team members. Work and life balance could be revealed through items about employee work balance, life balance, and the connection between work and life. Evaluation of social impact (value) could be evaluated through items about impact (value) of organization and employee personal social impact. All these items are interconnected and form a 5-dimension scale of meaningful work (see Table 5).

**Table 5:** Final version of the MSMW (dimensions and item description).

Dimensions	Number of items	Item description
Job design/environment (18 items)	5 items	*Opportunities*: teamwork or coworker assistance, learning and growth, independence and freedom, close friendships
	8 items	*Job performance*: clear instructions, methods, control, requirements and goals, task variety, decision making, depending on others, planning
	5 items	*Job security and feeling good*: possibility for multiple feedback, feeling of achievement, variety of knowledge and skills
Organizational commitment (17 items)	10 items	*Feelings about the organization and inside the organization*: happiness of belonging, accepting organizational problems as own, feeling proud, feeling as apart of organization, emotionally connected
	7 items	*Organizational loyalty and connection with the organization:* personal loyalty, social, emotional and economic loyalty, mission to believe in and corresponding values, personal meaning, care for organization
Meaningful Leadership (21 items)	3 items	*Relation with own supervisor:* standards and expectations, own contribution to performance, level of own contribution
	5 items	*Personal goal management:* establishing specific goals, determination toward own goals, monitoring own performance, reflection on the objectives and aspirations, self-evaluation
	9 items	*Teamwork:* teamwork skills, group encouragement for setting goals and giving ideas, collegial decision making, considering team ideas even if there is disagreement, helping team, explanation of decisions and actions, showing concern for team’s well-being and success, equality of team members
	4 items	*Relation with own team:* training team members, showing good examples, developing good relations, caring for team members
Work-life balance (14 items)	5 items	*Work balance:* satisfaction at work, time pressure for task completion, correspondence of time and energy to task completion, own value at work, dealing with problems
	5 items	*Life balance:* time for leisure and holidays, feelings about the future, energy for spare activities and tasks, feeling good, time for non-work activities
	4 items	*Work and life connection:* healthy work-life balance, personal life affecting work and vice versa, job gives energy for personal activities
Social impact (value) (10 items)	3 items	*Impact (value) of organization:* personal contribution to the continuous prosperity, impact on people outside the organization, promoting greater good.
	7 items	*Personal social impact:* impact on people and things through work, awareness of the beneficial ways of work, positive impact on customers and other parties of interest, value of personal contributions, consciousness of the positive impact, personal work benefits to others.

## 7 Ethics procedures

Data protection and non-disclosure agreement was signed between the authors and the organization. The agreement states that the data can be publicly presented only in this publication after receiving the approval of the organization’s representatives. Publicly available data that is representative of the analyzed dataset can be used to apply the methodology described in the article. All studies included in this construction and validation process adhered to ethical guidelines and data anonymity and confidentiality in accordance to the requirements set by the Office of Ombudsperson for Academic Ethics and Procedures of the Republic of Lithuania (Guidelines for the Assessment of Compliance with Research Ethics, 2020).

## 8 Discussion

### 8.1 Implications for theory

The series of studies presented through this paper on the MSMW construction and validity contributes conceptually to empirical research of meaningful work in companies and proposes several theoretical implications. *First*, the series of studies are focused on the construction of the meaningful work scale and its validity. Meaningful work as a concept embraces various dimensions such as work engagement, commitment, and job satisfaction, including citizenship behaviors, life meaning and overall life satisfaction (Allan et al., 2019; Hu and Hirsh, 2017). The latter dimensions are reflected through the MSMW. Sandoghar and Bailey (2023) specifically discuss the lack of clarity of the definition MW results from the several disciplines (philosophical business ethics, occupational and organizational psychologists, etc.) approaching it. While our scale is not to be used as a refined definition of meaningful work, it does intersect the major dimensions of the construct including *Job design/environment, Meaningful Leadership, Organizational Commitment, Work and Life Balance*, and *Social Impact*. Related to that, the multidisciplinary nature of meaningful work and the MSMW ensures that the construct may be analyzed in several contexts including business management, occupational psychology, ethics, human resource management, organizational management and ethics. *Second*, the proposed scale is broad and comprehensive, therefore, researchers may test the selected dimensions as separate in those cases when the full-scale model is not vital in certain business situations. *Third*, the proposed meaningful work scale would be useful for double-level testing inside organizations. Such an approach allows researchers and experts to test the meaningful work in two hierarchical levels (employee-employer) as different sources which may supply overall data for the detailed analysis of meaningful work. Then, the scale’s applicability to these different sources ensures the bridging of the different levels of institutions, businesses and organizations. Therefore, the proposed scale and its validation contributes to the existing theoretical background by highlighting the proposed meaningful work dimensions.

### 8.2 Implications for practice

The results suggest that the proposed meaningful work scale is valid and may be applied with businesses, and raise several practical implications. *First*, the proposed MSMW includes 5 important interconnected dimensions. Therefore, research determining the level of impact of each dimension on other dimensions should be conducted. *Second*, most research is focused on the benefits of employees, and how employers may benefit from employees’ sense of meaningful work (employee meaningful work makes the work environment better, increases turnover, decreases personnel changes, etc.) (Kim, 2023; Rabiul et al., 2023). Often, meaningful work research highlights employees’ potential moral aspirations; however, when analyzing the employer’s context, an employer’s potential moral obligation is most often highlighted, thus, eliminating the collegiality between employer and employee (Michaelson et al., 2014). Additionally, previous research indicates that employers may seek to control the existential domain of their employees, and may try to manipulate their meaningfulness for performative intent (Bailey et al., 2017). The lack of research on how the employers evaluate and experience meaningful work creates a niche for further research. *Third*, there is a trend of quantitative research related to meaningful work (Tan et al., 2023). Considering the multidimensionality and subjectivism within meaning-making, research combining qualitative and quantitative methods would provide broader and deeper situation analysis of meaningful work inside organizations. *Fourth*, most research is instantaneous and is not oriented toward longitudinal research that would create conditions to observe organizational and employee personal change in the context of meaningful work after certain interventions were made (Iždonaitė-Medžiūnienė and Batuchina, 2023). *Fifth*, the MSMW is oriented toward the personal experiences of employees eliminating professional interests, organizational policy, structure, culture, and values (Iždonaitė-Medžiūnienė and Batuchina, 2023). Therefore, further research including these aspects would be beneficial for the evolution of the proposed multidimensional meaningful work scale.

### 8.3 Limitations and future research

The research data analysis has several limitations that are significant for specifying the research context and contents. *First*, the research is limited to the meaningful work scale constructed from 5 subscales (job design/environment, leadership, organizational commitment, work and life balance, and social impact), while other scales include other dimensions. *Second*, although the research sample corresponded to the proposed ratio per item, it should be viewed as taking into consideration the context of one geographic area (Lithuania) and specific working area (construction). Research of meaningful work is also relevant to other cultural contexts (across countries), which may determine different experiences and results. Saulius and Malinauskas (2025, p. 13) reason that generally cultures shape emotion goals and its state of being, e.g., “East Asian cultures favor calmness, linking it to better adaptation, whereas Western cultures favor excitement, linking it to influence” which may have relation to the perception of a different worldview and many work-life processes that shape their conception of meaningful work and life. Further, previous research states that university students at their workplaces regulate their emotions in response to social challenges associated with personal priorities, work and communication, teamwork, and various types of collaboration (Saulius and Malinauskas, 2024) which in turn tend to have a strong impact on their meaningful work experience. Therefore, future studies should validate the scale with a diverse sample from across other cultures and contexts to ensure generalization of the results. It is important to have diversity in samples. The authors of this study will continue validating, translating, adapting and applying the current scale in other European and non-European countries. *Third*, emphasis has been placed on the relationship within an employee and organization to study meaningful work showing the impact of employee meaningful work on organization via job, organization and social contexts. However, there can be other factors such as an employee’s personal sense of meaningful work at an individual level (including gender, religious and spiritual beliefs, and generational identity), employee communicability and prosocial ability level. Related to the latter, research has demonstrated the importance of recognizing and holistically measuring meaningful work with contextual factors such as gender, age, and religious and spiritual beliefs (Burbano et al., 2024; Hoole and Bonnema, 2015; Vveinhardt and Deikus, 2023a). In addition, Lysova et al. (2019) propose that people who are pro-socially motivated may experience more meaningful work in their jobs. Future studies should seek to cover these factors, also including gender aspects. *Fourth*, the limitation of the research lies within the organizational context, while experience of meaningful work across different industries or sectors would be an implication for further research considering profit and non-profit oriented organizations, or educational or medical organizations, which may confidently provide a reinforced sense of meaningful work for employees. *Fifth*, further research should include the other stages as described by Lamm et al. (2020): external structure validity completion, and consequential validity implementation. *Sixth*, the other limitation of this research is its disposable nature. Future research opportunities should include the need for longitudinal data collection (pre-test and post-test): after completing the meaningful work study inside any organization, recommended actions should be implemented, and repetitive study should be completed to assess the change in meaningful work data. *Finally*, organizations may benefit from experimentally applying this measure including a linear approach (pre-test measurement through the MSMW, strategic intervention in the organization, post-test measurement of the MSMW). Strategic interventions may differ on which topics and issues they may tackle. Vveinhardt and Deikus (2023b) explored religious strategies to be implemented in the workplace for workplace mobbing. Others have explored gender-based interventions for gender equity in the workplace (Tricco et al., 2024). While generation-based interventions are scarce, strategic interventions for meaningful work must be explored and applied based on the context of the organization including relevant factors such as male–female employee ratio, religious and spiritual beliefs. In turn, the combination of these aspects may have a realistic impact on organizations as well as on the research surfacing through these organizations and the use of the MSMW.

## 9 Conclusion

The research and validation processes have created several important theoretical and practical aspects. Thus, based on the scientific review and performed validation process of the meaningful work scale several conclusions can be formulated. Firstly, the meaningful work idea is a very subjective concept and really depends on the beliefs, ideas, goals and aims of the employee. Here lies a challenge of definition and understanding of meaningful work. Secondly, the importance of meaningful work goes beyond job satisfaction, performance and commitment to the organization’s ideas. However, by prioritizing meaningful work organizations can maximize the productivity and well-being of the employees. Thirdly, modern processes such as digitalization, always on working culture, hybrid working conditions might have an impact on the understanding of meaningful work. Moreover, meaningful work is a complex and multidimensional concept, also depending on the different job factors and circumstances, and as such should be studied alongside concepts such as leadership style, organizational structure, and demographic factors.

From the practical point of view, a deep and detailed validation process, which included both qualitative and quantitative methods, showed that the constructed multidimensional meaningful work questionnaire is a reliable instrument, while the implemented stages offered the instrument added value, rigor and quality. Ultimately, the proposed tool can be used as a valuable tool for measuring meaning at work, identifying and detecting the areas for individual and organizational improvement with the goal of increasing overall employee satisfaction, commitment, and performance.

## Data Availability

The datasets presented in this article are not readily available because data protection and non-disclosure agreement was signed between the authors and the organization. The agreement states that the data can be publicly presented only in this publication after receiving the approval of the organization’s representatives. Publicly available data that is representative of the analyzed dataset can be used to apply the methodology described in the article. However, they can not be shared, anonymized or otherwise as this was a restriction imposed by third parties involved in this research (e.g., participating companies). Requests to access the datasets should be directed to aleksandra.batuchina@dest.smk.lt.
